# The efficacy of red and blue light-emitting diode phototherapy combined with oral minocycline for acne conglobata: a retrospective cohort study

**DOI:** 10.3389/fmed.2025.1708077

**Published:** 2026-01-12

**Authors:** Yonghong Hao, Xiaofang Zou, Yixuan Gao, Liyuan Xing, Chengxin Li

**Affiliations:** 1 Department of Dermatology, Chinese PLA General Hospital, Beijing, China; 2 Department of Burns and Plastic Surgery, First Affiliated Hospital of Chinese PLA General Hospital, Beijing, China; 3 Department of Dermatology, First Affiliated Hospital of Chinese PLA General Hospital, Beijing, China

**Keywords:** acne conglobata, phototherapy, light-emitting diode therapy, minocycline, retrospective study

## Abstract

**Objective:**

Acne conglobata (AC) is a severe inflammatory skin condition with limited therapeutic options that can provide rapid and sustained remission. This retrospective cohort study aimed to evaluate the clinical efficacy and safety of a combination therapy of twice-weekly red-blue light-emitting diode (LED) phototherapy with a shortened 4-week course of oral minocycline, compared to a conventional polypharmacy regimen for AC.

**Methods:**

We analyzed clinical data from 28 outpatients diagnosed with AC. The study group (*n* = 15) received LED phototherapy (640 nm red and 460 nm blue light) and oral minocycline (100 mg/day for 4 weeks). The control group (*n* = 13) was treated with a heterogeneous conventional regimen, including oral isotretinoin and/or extended minocycline (8 weeks) plus topical agents. Efficacy outcomes, including Symptom Score Reduction Index (SSRI), Global Acne Grading System (GAGS), and Pruritus Visual Analog Scale (P-VAS), were assessed by blinded dermatologists at baseline and over 8 weeks. Safety and tolerability were also evaluated.

**Results:**

Baseline characteristics were comparable between groups. The LED-minocycline combination resulted in significantly superior and more rapid clinical improvement. At week 8, 100% of patients in the study group achieved cure (SSRI ≥90%), versus 0% in the control group (*p* < 0.001). The mean SSRI in the study group was 92.1 ± 2.37% compared to 23.1 ± 4.35% in the control group (*p* < 0.001). The study group also showed significantly lower post-treatment GAGS scores (7.01 ± 2.01 vs. 9.03 ± 2.36, *p* = 0.022) and P-VAS scores (1.2 ± 0.6 vs. 4.8 ± 1.1, *p* < 0.001). Adverse events were mild and transient, with no significant difference in the incidence of skin irritation between groups (93.3% vs. 84.6%, *p* = 0.583).

**Conclusion:**

Combining red-blue LED phototherapy with a 4-week course of minocycline is a rapid, highly effective, and safe therapeutic strategy for AC, outperforming conventional polypharmacy while halving the duration of systemic antibiotic exposure. Despite the inherent limitations of a retrospective design, these findings support the integration of LED therapy to optimize the management of severe acne.

## Introduction

Acne conglobata (AC) represents a severe, chronic, and often disfiguring subtype of nodulocystic acne, characterized by inflammatory nodules, abscesses, interconnecting sinus tracts, and resultant scarring (1). The pathogenesis is multifactorial, involving follicular hyperkeratinization, sebum overproduction, colonization by *Cutibacterium acnes* (formerly *Propionibacterium acnes*), and complex inflammatory and immunological responses (2). Affecting approximately 20% of adolescents and young adults with moderate-to-severe acne (3), AC can lead to significant psychological distress, including anxiety, depression, and social withdrawal, in addition to permanent physical scarring (4, 5).

Standard therapeutic guidelines for severe acne recommend systemic agents, including oral antibiotics (e.g., tetracyclines) and oral isotretinoin (6). While highly effective, these treatments are associated with potential adverse effects, require prolonged administration, and oral antibiotics contribute to the growing concern of antimicrobial resistance (7). Consequently, there is a compelling need for effective, safe, and non-invasive adjunctive or alternative therapies that can accelerate clinical response, reduce reliance on long-term systemic medication, and improve overall outcomes.

Light-emitting diode (LED) phototherapy has emerged as a promising modality in dermatology. Blue light (typically 415–470 nm) exerts antimicrobial effects by activating endogenous porphyrins within *C. acnes*, leading to bacterial destruction (8). Red light (typically 630–660 nm) penetrates deeper into the dermis, exerting potent anti-inflammatory effects by modulating cytokine production and promoting tissue repair (9). A recent meta-analysis has substantiated the efficacy of combined blue and red light therapy for inflammatory acne, demonstrating significant reductions in lesion counts with a favorable safety profile (10). However, robust data on its specific efficacy for the most severe forms, such as AC, and particularly on optimized combination protocols with systemic antibiotics, remains limited. A key clinical question is whether adjunctive LED therapy can allow for a shortened duration of systemic antibiotics, thereby minimizing associated risks.

This study was therefore designed to retrospectively investigate the real-world effectiveness of combining twice-weekly red and blue LED phototherapy with a 4-week course of oral minocycline for the treatment of AC. We hypothesized that this combination would achieve a more rapid and superior clinical remission compared to a conventional, longer-duration polypharmacy regimen, thereby offering a valuable strategy to optimize the management of this challenging condition.

## Materials and methods

### Study design and ethical conduct

This study was a retrospective cohort analysis of patient data collected from the First Affiliated Hospital of Chinese PLA General Hospital between January 2015 and January 2021. The study was conducted in accordance with the Declaration of Helsinki. The protocol was approved by the Ethics Committee of the First Affiliated Hospital of Chinese PLA General Hospital. Written informed consent was obtained from all participants for the use of their clinical data and images for research purposes. This report adheres to the Strengthening the Reporting of Observational Studies in Epidemiology (STROBE) guidelines (11).

### Subjects

A total of 28 outpatients diagnosed with facial AC were included. Patient allocation into the study group (*n* = 15) or control group (*n* = 13) was based on the documented treatment plan prescribed by the attending physician. This non-randomized allocation was influenced by real-world clinical factors, including patient preference for non-invasive treatments, previous treatment history, and the ability to cover the cost of the LED therapy course. We explicitly acknowledge this as a significant limitation introducing potential selection bias, and its impact on the interpretation of outcomes is discussed.

### Inclusion and exclusion criteria

Inclusion Criteria were: ① Clinical diagnosis of acne conglobata according to the 2019 Chinese Guidelines for Acne Treatment (12); ② Involvement of the full face; ③ Presence of at least 6 cystic lesions greater than 1 cm in diameter; ④ No concurrent systemic or topical acne medications other than those prescribed in the study protocol; ⑤ No history of photosensitivity or known allergies to study medications; ⑥ No other acne treatments in the preceding 3 months; ⑦ Age between 18 and 38 years; ⑧ Disease duration at presentation between 9 and 21 days.

Exclusion Criteria included: ① Pregnancy, lactation, or active menstruation at the time of enrollment; ② Severe systemic or psychiatric comorbidities; ③ History of keloid formation or significant post-inflammatory pigmentary alterations; ④ Documented non-compliance with treatment protocols; ⑤ Presence of facial wounds, active herpes simplex, or warts; ⑥ Long-term use of corticosteroids/immunosuppressants or recent X-ray therapy; ⑦ Discontinuation of treatment due to adverse events or worsening of the condition.

### Treatments

#### Study group (LED + minocycline)

Patients in this group (*n* = 15) received a combination of LED phototherapy and a 4-week course of oral minocycline.

##### Red and blue light irradiation therapy

Patients were treated twice weekly for 8 weeks (total 16 sessions) using an LED-IB light source device (Shenzhen Prolumen Technology Co., Ltd.) positioned 15 cm from the skin. The treatment consisted of red light (wavelength 640 ± 10 nm; irradiance 100 mW/cm^2^) for 20 min, followed by blue light (wavelength 460 ± 10 nm; irradiance 100 mW/cm^2^) for 10 min. These parameters are consistent with established protocols for acne treatment that have demonstrated both antibacterial and anti-inflammatory efficacy (13). The irradiance was based on the nominal output of the device, and operators ensured uniform facial coverage by adjusting the panel’s position as needed.

##### Systemic and adjunctive therapy

Patients were prescribed oral minocycline (50 mg, twice daily) for a fixed duration of 4 weeks. They were counseled on sun protection due to minocycline’s potential for photosensitivity (14). For supportive care, which was identical in both groups, a post-LED hydrogen peroxide solution was applied for 15 min for gentle debridement and disinfection, followed by a daily Koligen® human collagen moisturizing mask to enhance hydration and support skin barrier function, thereby managing potential treatment-induced irritation.

#### Control group (conventional therapy)

Patients in the control group (*n* = 13) received a conventional polypharmacy regimen for 8 weeks. The regimen was heterogeneous, as detailed in Supplementary Table 1. The main components were:

*Oral medication*: This typically included oral isotretinoin (50 mg/day) and/or a full 8-week course of minocycline (100 mg/day). We acknowledge that the historical concomitant use of isotretinoin and minocycline in some patients is contraindicated due to an increased risk of pseudotumor cerebri (15). This reflects a past clinical practice and is a significant limitation of this historical cohort’s data.

*Topical medication*: This included once-daily applications of benzoyl peroxide gel, adapalene gel, and fusidic acid gel.

*Adjunctive therapy*: Similar to the study group, patients used the daily Koligen® collagen mask for hydration and skin barrier support.

### Efficacy and safety assessment

Two dermatologists, blinded to treatment allocation, assessed clinical efficacy at baseline and weekly for 8 weeks using standardized high-resolution photographs. The assessment criteria were pre-specified based on established acne scoring systems.

1. Symptom score (SS) and SSRI: Lesions were counted and graded to calculate a total SS. The SS Reduction Index (SSRI) was derived as [(pretherapy SS – posttherapy SS) / pretherapy SS] × 100%. Efficacy was categorized as: cure (SSRI ≥ 90%), substantial improvement (60% ≤ SSRI < 90%), improvement (20% ≤ SSRI < 60%), and ineffectiveness (SSRI < 20%).
2. Global acne grading system (GAGS): This standardized system was used to assess overall acne severity (16).
3. Pruritus visual analog scale (P-VAS): Patients rated their average itch intensity over the past week on a 10-point scale.
4. Dermatology life quality index (DLQI): At baseline and week 8, patients completed the DLQI questionnaire, a validated 10-item survey to assess the impact of their skin condition on quality of life. Scores range from 0 to 30, with higher scores indicating greater impairment (17).
5. Patient-reported tolerability: At week 8, patients rated the overall tolerability of their regimen on a 4-point Likert scale.

Adverse events were recorded at each visit. All patients were followed for 12 months post-treatment to assess recurrence.

### Statistical analysis

Data were analyzed using SPSS software (v26.0). Continuous variables were expressed as mean ± standard deviation (SD) and compared using independent t-tests or Mann–Whitney U tests. Within-group changes were analyzed with paired t-tests or Wilcoxon signed-rank tests. Categorical data were compared using the Chi-square or Fisher’s exact test. A two-tailed *p*-value <0.05 was considered statistically significant. Given the small sample sizes and the use of some non-parametric tests, the assumptions for parametric tests were carefully considered, and non-parametric alternatives were used where distributions were not normal to ensure the robustness of the findings.

## Results

### Baseline characteristics

The study and control groups were well-matched at baseline. There were no statistically significant differences in age, sex, BMI, allergy history, or disease duration (all *p* > 0.05; Table 1). Initial disease severity was also comparable, with no significant difference in baseline GAGS scores (*p* = 0.931), P-VAS scores (*p* = 0.672), or DLQI scores (22.5 ± 3.1 vs. 23.1 ± 2.8, *p* = 0.591; Tables 2–4).

**Table 1:** The comparison of general information in two groups.

Index	Study group (*n* = 15)	Control group (*n* = 13)	Statistic	*p*-value
Gender (*n*, %)			*χ*^2^ = 0.165	0.684
Female	7 (46.7%)	8 (61.5%)		
Male	8 (53.3%)	5 (38.5%)		
Age (year, mean ± SD)	20.1 ± 2.53	19.0 ± 2.20	t = −1.267	0.216
Disease duration at baseline (day, mean ± SD)	16.7 ± 4.65	14.0 ± 4.80	t = −1.525	0.140
BMI (kg/m^2^, mean ± SD)	22.6 ± 2.50	22.4 ± 3.74	t = −0.151	0.881
Allergic history (*n*, %)				0.433^#^
+	4 (26.7%)	6 (46.2%)		
−	11 (73.3%)	7 (53.8%)		

BMI, body mass index; SD, standard deviation. ^#^Fisher’s exact test was used.

**Table 2:** Comparison of GAGS scores between two groups (mean ± SD).

Group	Before treatment	After 8 weeks of treatment
Study group (*n* = 15)	28.21 ± 5.21	7.01 ± 2.01
Control group (*n* = 13)	28.03 ± 5.68	9.03 ± 2.36
*t*-statistic	−0.087	2.447
*p*-value	0.931	0.022

**Table 3:** Comparison of pruritus visual analog scale (P-VAS) scores between two groups (mean ± SD).

Group	Before treatment	After 8 weeks of treatment
Study Group (*n* = 15)	7.9 ± 1.1	1.2 ± 0.6
Control Group (*n* = 13)	8.1 ± 1.3	4.8 ± 1.1
*t*-statistic	0.427	10.145
*p*-value	0.672	<0.001

**Table 4:** Comparison of dermatology life quality index (DLQI) scores (mean ± SD).

Group	Before treatment	After 8 weeks of treatment	Mean change (baseline–week 8)	*p*-value (for change)
Study Group (*n* = 15)	22.5 ± 3.1	4.7 ± 2.5	−17.8 ± 2.9	<0.001
Control Group (n = 13)	23.1 ± 2.8	13.6 ± 3.3	−9.5 ± 3.1	
*p*-value (between groups)	0.591	<0.001	–	–

### Clinical efficacy outcomes

**Figure 1:**
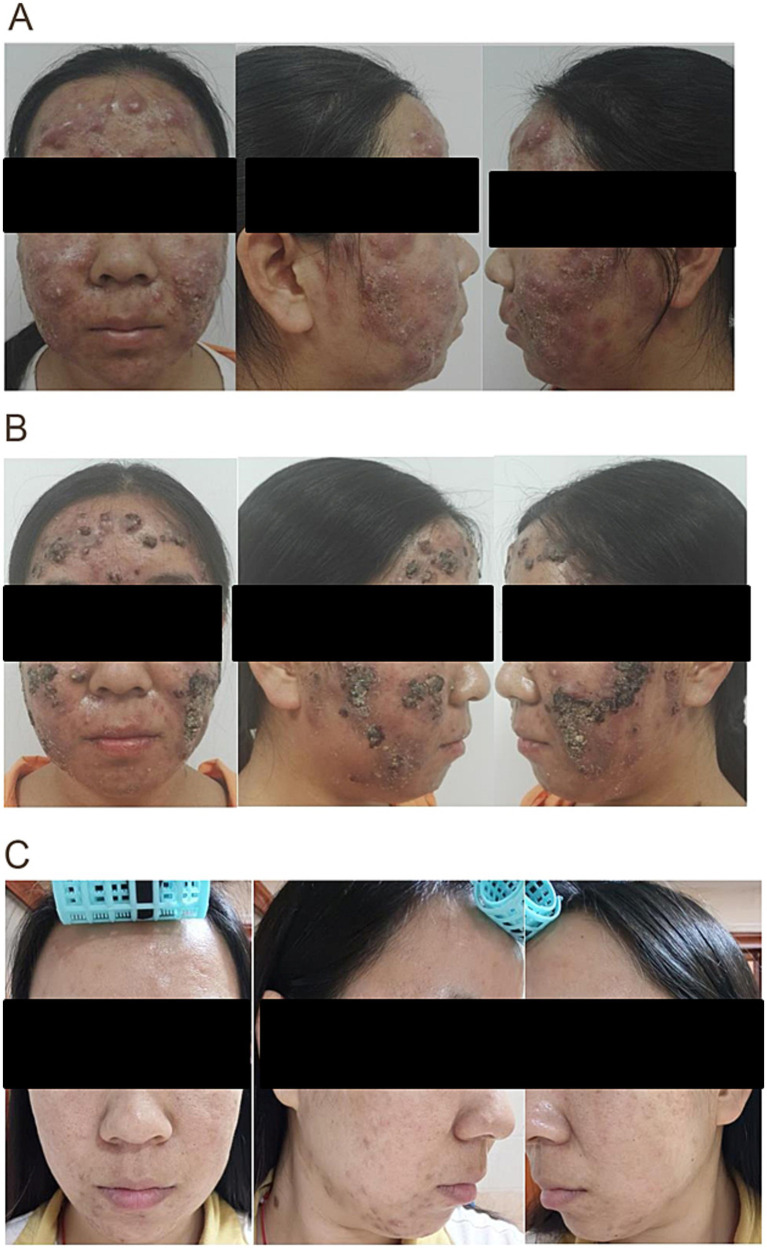
Clinical improvement of acne conglobata in the study group. Photographs of a 19-year-old female patient from the study group showing severe inflammatory nodules and cysts at baseline **(A)**; marked reduction in inflammation and pustule formation after 1 week of treatment **(B)**; and near-complete resolution with residual post-inflammatory erythema at the 8-week endpoint **(C)**. Written informed consent was obtained from the patient for the publication of these images.

The LED-minocycline regimen demonstrated a significantly faster and more profound clinical response. By week 8, 100% of patients (15/15) in the study group achieved ‘cure’ status (SSRI ≥90%), whereas no patients in the control group reached this endpoint (*p* < 0.001; Table 5). This marked improvement is visually represented in Figure 1.

**Table 5:** Comparison of therapeutic efficacy based on SSRI categories.

Time point	Study group (*n* = 15)				Control group (*n* = 13)				*p*-value (Intergroup)^#^
	Cure (≥90%)	Excellent (60–89%)	Good (20–59%)	Poor (<20%)	Cure (≥90%)	Excellent (60–89%)	Good (20–59%)	Poor (<20%)	
Week 1	0 (0%)	0 (0%)	15 (100%)	0 (0%)	0 (0%)	0 (0%)	0 (0%)	13 (100%)	<0.001*
Week 8	15 (100%)	0 (0%)	0 (0%)	0 (0%)	0 (0%)	0 (0%)	7 (53.8%)	6 (46.2%)	<0.001*

Data presented as *n* (%). ^#^Comparison between study and control groups using Fisher’s exact test. *Statistically significant.

**Figure 2:**
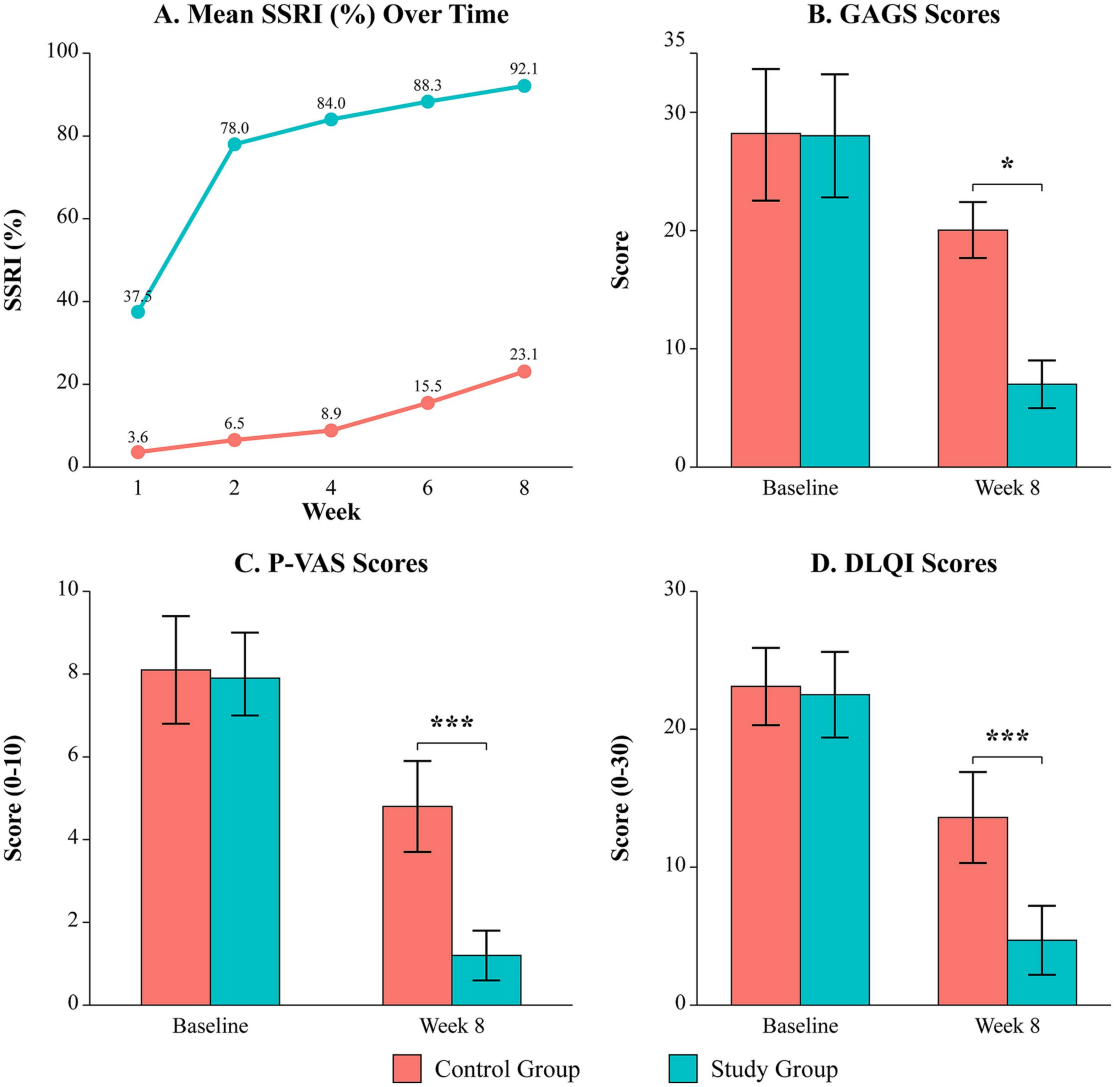
Longitudinal comparison of efficacy and quality of life outcomes. **(A)** Mean symptom score reduction index (SSRI, %) over 8 weeks. **(B)** Mean global acne grading system (GAGS) scores at baseline and week 8. **(C)** Mean pruritus visual analog scale (P-VAS) scores at baseline and week 8. **(D)** Mean dermatology life quality index (DLQI) scores at baseline and week 8. Data are presented as mean ± SD. *p < 0.05, **p < 0.001 vs. control group.

Longitudinal SSRI analysis showed a rapid divergence in efficacy. The study group’s mean SSRI was 37.5 ± 3.85% at week 1, reaching 92.1 ± 2.37% by week 8, compared to 3.62 ± 1.26% and 23.1 ± 4.35% in the control group (*p* < 0.001 at all timepoints; Table 6; Figure 2A).

**Table 6:** Comparison of symptom score reduction index (SSRI, %) (Mean ± SD).

Time point	Study group (*n* = 15)	Control group (*n* = 13)	*t*-statistic	*p*-value
Week 1	37.5 ± 3.85	3.62 ± 1.26	−32.172	<0.001*
Week 2	78.0 ± 5.28	6.54 ± 0.66	−51.971	<0.001*
Week 4	84.0 ± 4.31	8.85 ± 0.80	−66.236	<0.001*
Week 6	88.3 ± 3.35	15.5 ± 1.61	−74.716	<0.001*
Week 8	92.1 ± 2.37	23.1 ± 4.35	−50.992	<0.001*

*Statistically significant.

At the 8-week endpoint, mean GAGS scores (7.01 ± 2.01 vs. 9.03 ± 2.36, *p* = 0.022) and P-VAS scores (1.2 ± 0.6 vs. 4.8 ± 1.1, *p* < 0.001) were significantly lower in the study group (Tables 2, 3; Figures 2B,C). Furthermore, the study group experienced a significantly greater improvement in quality of life, with the mean DLQI score decreasing by 17.8 points compared to a 9.5-point decrease in the control group (*p* < 0.001; Table 4; Figure 2D).

### Safety and tolerability

Both treatment regimens were generally well-tolerated. The most frequent adverse event was transient skin irritation, with no significant difference between groups (93.3% vs. 84.6%, *p* = 0.583). Dizziness was also comparable (20.0% vs. 15.4%, *p* = 1.000; Table 7). No severe adverse events occurred. Patient-reported tolerability was high in both groups (*p* = 0.235; Table 8).

**Table 7:** Comparison of adverse events in the two groups.

Adverse event	Status	Study group (*n* = 15)	Control group (*n* = 13)	*p*-value^#^
Dizziness	+	3 (20.0%)	2 (15.4%)	1.000
	−	12 (80.0%)	11 (84.6%)	
Skin irritation	+	14 (93.3%)	11 (84.6%)	0.583
	−	1 (6.7%)	2 (15.4%)	

Data presented as *n* (%). ^#^Fisher’s exact test was used.

**Table 8:** Patient-reported tolerability at week 8.

Tolerability rating	Study group (*n* = 15)	Control group (*n* = 13)	*p*-value^#^
Excellent	5 (33.3%)	3 (23.1%)	0.235
Good	8 (53.3%)	7 (53.8%)	
Fair	2 (13.3%)	3 (23.1%)	
Poor	0 (0%)	0 (0%)	

Data presented as *n* (%). ^#^Fisher’s exact test was used.

### Long-term follow-up

During the 12-month follow-up, the study group showed a more stable remission. Recurrence of papules induced by lifestyle factors was significantly lower in the study group (60.0%) compared to the control group (100.0%) (*p* = 0.018; Table 9).

**Table 9:** Comparison of relapse during 12-month follow-up.

Trigger for relapse	Status	Study group (*n* = 15)	Control group (*n* = 13)	p-value^#^
Lifestyle (Stay up late/spicy diet)	+	9 (60.0%)	13 (100%)	0.018*
	−	6 (40.0%)	0 (0%)	
Menstrual Period (Females only)	+	7/7 (100%)	8/8 (100%)	1.000
	−	0/7 (0%)	0/8 (0%)	

Data presented as *n* (%). ^#^Fisher’s exact test was used. *Statistically significant.

## Discussion

This study provides compelling real-world evidence that combining twice-weekly red-blue LED phototherapy with a shortened 4-week course of oral minocycline is a highly effective and rapid treatment for AC. The study group achieved a 100% cure rate within 8 weeks, a result not seen in the control group. This aligns with the synergistic mechanisms of the combined therapy: blue light provides bactericidal effects against *C. acnes*, while red light offers potent anti-inflammatory action, which, when combined with the systemic effects of minocycline, creates a powerful multi-pronged attack on the pathophysiology of AC (8, 18).

A key finding of this study is the clinical implication of the shortened minocycline course. Achieving superior results with only 4 weeks of minocycline, compared to the 8-week systemic therapies in the control arm, addresses the critical goal of reducing systemic antibiotic exposure (19). This indicates that LED phototherapy can act as a potent “antibiotic-sparing” adjunct, accelerating the therapeutic response and mitigating risks like microbial resistance and systemic side effects. This concept is supported by studies where adjunctive photodynamic therapy has been shown to allow for effective, truncated courses of oral antibiotics in moderate-to-severe acne (20, 21).

The rapid improvement and superior reduction in pruritus and quality of life impairment (DLQI) in the study group are clinically significant. A faster resolution of symptoms can dramatically improve patient adherence and psychological well-being (5). The control group’s slower progress may be partly explained by the known delayed onset for oral isotretinoin, which can take several weeks to months for maximal effect (22).

The safety profiles were comparable, with manageable transient skin irritation being the most common side effect in both groups. The high incidence in the study group (93.3%) is likely a cumulative effect of the LED sessions and the post-treatment hydrogen peroxide application, rather than LED therapy alone, which is generally well-tolerated (10). The rate was not significantly different from the control group, which used known irritants like benzoyl peroxide and adapalene.

This study has several significant limitations. Firstly, its retrospective nature and non-randomized allocation introduce a high risk of selection bias. Secondly, the small sample size limits statistical power. Thirdly, the control group was heterogeneous and included a contraindicated combination of isotretinoin and minocycline in some patients. This reflects a historical clinical practice but is not an ideal comparator. A more appropriate control would have been a group receiving minocycline for an identical duration without LED. Lastly, while outcome assessors were blinded, patients and treating physicians were not, which could introduce bias.

## Conclusion

In conclusion, despite its inherent limitations, this retrospective study suggests that combining twice-weekly red-blue LED phototherapy with a 4-week course of oral minocycline can achieve rapid and complete remission in patients with AC. This approach appears superior to conventional polypharmacy in efficacy, speed of response, and quality of life improvement, while allowing for a 50% reduction in the duration of systemic antibiotic therapy. These findings highlight the potential of integrated phototherapy as a powerful strategy in managing severe inflammatory acne. Prospective, multicenter, randomized controlled trials are imperative to confirm these promising results.

## Data Availability

The original contributions presented in the study are included in the article/Supplementary material, further inquiries can be directed to the corresponding author.
