# Supplementary material for: The efficacy of red and blue light-emitting diode phototherapy combined with oral minocycline for acne conglobata: a retrospective cohort study
Source: Front Med (Lausanne). 2026 Jan 12;12:1708077. doi: 10.3389/fmed.2025.1708077 (PMC12832268; doi:10.3389/fmed.2025.1708077)
Supplement: Supplementary file 1 [file Table_1.docx]

**Supplementary Table 1. Detailed Breakdown of Heterogeneous Treatments in the Control Group (n=13)**

| **Subgroup ID** | **Number of Patients (n)** | **Oral Medications (Duration: 8 weeks)** | **Topical Medications (Duration: 8 weeks)** |
| --- | --- | --- | --- |
| C1 | 5 | Isotretinoin 50mg/day + Minocycline 100mg/day + Zinc Glycyrrhizinate | Benzoyl Peroxide, Adapalene, Fusidic Acid |
| C2 | 5 | Minocycline 100mg/day + Zinc Glycyrrhizinate | Benzoyl Peroxide, Adapalene, Fusidic Acid |
| C3 | 3 | Isotretinoin 50mg/day + Zinc Glycyrrhizinate | Benzoyl Peroxide, Adapalene, Fusidic Acid |
|  | | | |

Note: This table illustrates the heterogeneity of the control arm, reflecting real-world prescribing variations for severe acne. The concomitant use of isotretinoin and minocycline in subgroup C1 is acknowledged as a deviation from current safety guidelines and represents a significant limitation of the data from this historical cohort. All patients in the control group used a daily collagen mask.
